# Editorial: Synaptic Plasticity for Neuromorphic Systems

**DOI:** 10.3389/fnins.2016.00214

**Published:** 2016-05-19

**Authors:** Christian G. Mayr, Sadique Sheik, Chiara Bartolozzi, Elisabetta Chicca

**Affiliations:** ^1^Chair of Highly-Parallel VLSI-Systems and Neuromorphic Circuits, Technische Universität DresdenDresden, Germany; ^2^BioCircuits Institute, University of California, San DiegoSan Diego, CA, USA; ^3^iCub Facility, Istituto Italiano di TecnologiaGenova, Italy; ^4^Cognitive Interaction Technology - Center of Excellence, Faculty of Technology, Bielefeld UniversityBielefeld, Germany

**Keywords:** synaptic plasticity, neuromorphic engineering, memristive plasticity, plasticity circuits, digital plasticity, high-density plasticity, plasticity for sensor data, learning feature extraction

Brain plasticity serves animals in a wide range of vital functions. It assists them in adapting their behavior to the surroundings, in learning new strategies for optimizing a certain reward-seeking policy for their survival or in adjusting motor activity through sensory feedback. Thus, plasticity is an essential ingredient for building artificial autonomous systems that can cope with the real world. In order to build these systems, neuromorphic design labs actively investigate and develop various circuit implementations of plasticity. This research topic collects a comprehensive snapshot of this work. A number of manuscripts published in this topic study the interplay between stochasticity and plasticity (Afshar et al.; Bill and Legenstein; Lagorce et al.; Qiao et al.). Plasticity here acts in a stochastic fashion or extracts features from stochastic sensor data. The current push toward higher complexity/scale in neuromorphic devices can also be observed in plasticity implementations (Qiao et al.; Wang et al.; Noack et al.). Due to advantageous technology scaling and reproducibility, digital implementations of neuromorphic plasticity are gaining popularity (Galluppi et al.; Vogginger et al.). The collection of articles in this topic is rounded out by articles on plasticity in novel nano-scale technologies (Saighi et al.; Thomas et al.; Wang et al.; Bill and Legenstein).

## 1. Stochasticity and plasticity

One topic of interest in recent publications is the interaction between stochasticity and synaptic dynamics. Wang et al. introduces a stochastic synapse cell constructed with a memristor and two transistors. Bill and Legenstein show that stochastic synapses can provide graded responses from binary-valued synapses, aiding convergence in learning tasks. Stochasticity in conjunction with plasticity can also aid error tolerance. For instance, the stochastic synapse model of Bill and Legenstein can learn handwritten digits with high fidelity in the presence of significant device deviations and noise. The statistical inference in Afshar et al. actually uses deviations between individual dendrites. The visual feature extraction in Lagorce et al. operates on a high-dimensional projection of the input space through a recurrent neural network, benefitting from deviations across elements. A more conventional error-compensation approach is taken in Qiao et al., where a network counterbalances for deviations through a learned aggregate of individual neuronal responses.

## 2. Plasticity operating on sensor data

The above mentioned Qiao et al. and Lagorce et al. also represent examples of processing and plasticity operating directly on spiking input. In fact, typical tasks that would be amenable to a neuromorphic solution have traditionally used non-spiking input, such as image processing applications exclusively using image frames (Henker et al., 2007). Due to these incompatible representations (e.g., continuous time vs. discrete time, spikes vs. scalar values), there has not been much synergy between neuromorphic and traditional sensor processing, thus potentially missing some novel approaches in both fields. However, the two are growing closer together as sensors with spiking output are becoming more widely available in such diverse areas as vision (Delbruck, 2008) or audition (Liu et al., 2010). In addition to the plastic sensory processing in Qiao et al. and Lagorce et al., the statistical inference of Afshar et al. could also be employed for sensory processing, as it is geared toward the temporal patterns of multiple input spike trains.

## 3. Digital implementations of plasticity

Neuromorphic engineering was envisioned as analog VLSI circuits, due to the similarity between the current across CMOS devices in subthreshold and across neurons ion channels. However, digital circuits benefit significantly more from technology scaling and low-power advances in deep-submicron nodes, making them attractive for neuromorphic implementations. Specifically, plasticity allows fixed, reproducible-function digital circuits to add adaptability and variation to their behavior. Galluppi et al. present a framework for plasticity implementation on a programmable digital neuromorphic system, SpiNNaker. Vogginger et al. discuss a computational optimization of a powerful learning rule, outlining an efficient implementation in a synthesized or programmable digital neuromorphic system. Afshar et al. present an FPGA implementation of a novel neuron model and an accompanying learning rule optimized for digital circuits.

## 4. Large scale hardware for plasticity

Complex real-world applications demand large, computationally capable neural networks. Consequently, there is a drive toward large scale neuromorphic hardware with plasticity. The chip of Qiao et al. is currently one of the largest devices with on-chip plasticity (256 neurons, 128k synapses, 180 nm CMOS) that employs the original subthreshold design philosophy. Noack et al. present a switched capacitor implementation of short- and long-term plasticity in 28 nm CMOS that at 3.6 × 3.6 μ*m*^2^ is an order of magnitude smaller than any other plastic CMOS synapse. Other approaches to scaling include Wang et al., which uses a digital time-multiplexed circuit to compute Spike Timing Dependent Plasticity (STDP) for time-multiplexed analog neurons. For large-scale networks, topological considerations also play an increasing role, e.g., in terms of which signals a plasticity circuit needs access to (e.g., pre- or post-synaptic) (Noack et al., 2010). A neuron-synapse matrix arrangement seems the obvious choice, but the implementations in this topic explore a variety of other options.

## 5. Memristive plasticity

In terms of emerging technologies, the usage of nanoscale memristors for short- or long term plasticity has seen a large deal of interest since the pioneering work of Jo et al. (2010). Memristors inherently replicate aspects of synaptic plasticity and can combine plasticity, weight storage and weight effect in a single device. Saighi et al. gives an overview of recent developments in this area from a materials and neuromorphic perspective. Thomas et al. investigate tunnel junction based memristors that exhibit STDP-like plasticity. Wang et al. present a synaptic cell composed of memristor plus transistors which endows the synapse with stochastic learning capabilities. Bill and Legenstein introduce a model of an ideal stochastic memristor synapse and investigate its computational properties.

## 6. Summary

Synaptic plasticity is a crucial ingredient in neuromorphic hardware. It has the potential to contribute to many different fields, such as in the endeavor of building realistic brain models, in biohybrids where the hardware adapts to the biological counterpart or in the construction of truly cognitive systems. This research topic gives an overview of the state-of the art in plasticity circuit design and applications and outlines future research directions.

## Author contributions

CM, EC, CB, and SS all contributed to the compilation of plasticity works described in this editorial and to the writing of the editorial.

### Conflict of interest statement

The authors declare that the research was conducted in the absence of any commercial or financial relationships that could be construed as a potential conflict of interest.
